# A quick response, but not too much: Experienced mangabeys (*Cercocebus torquatus*) lose interest in contact call exchanges that do not respect this “conversational rule”

**DOI:** 10.1371/journal.pone.0310857

**Published:** 2024-11-13

**Authors:** Bastien Meunier, Virginie Durier, Arnaud Rossard, Alban Lemasson

**Affiliations:** 1 Université de Rennes, CNRS, Université de Normandie, EthoS (Ethologie Animale et Humaine)–UMR 6552, Rennes, France; 2 Institut Universitaire de France, Paris, France; University of Stirling, UNITED KINGDOM OF GREAT BRITAIN AND NORTHERN IRELAND

## Abstract

Several non-human primate species engage in vocal exchanges of contact calls, throughout the day in peaceful contexts. These vocal exchanges have been compared to human conversations because vocalizations are uttered in turn-taking: a temporal pattern where interlocutors minimize silences and avoid overlaps. But observing such a pattern in the spontaneous production of a species, as is the case with red-capped mangabeys (*Cercocebus torquatus*), is not enough to make it a rule. Another prerequisite is that the pattern is expected by the animal. Here, we conducted a playback experiment using the violation-of-expectation paradigm to test whether captive red-capped mangabeys react differently to usual *vs* unusual interactive temporal patterns. We played back vocal exchanges with usual minimized response time (0.5 sec), with unusual longer response time (1.5 sec) and with unusual call overlap to 12 adult captive male mangabeys. For each individual, we measured the occurrences and durations of head orientation toward the loudspeaker after the stimuli. The interest of individuals varied according to the vocal exchange temporal pattern in interaction with their age. Indeed, the older (and thus more socially experienced) an individual was, the less interested he became after an unusual vocal exchange, i.e. a vocal exchange with call overlap or with a delayed response time. These findings suggest that experience shapes attention towards more socially relevant situations, and thus that turn-taking can be qualified as a social rule.

## Introduction

Animals interact vocally with conspecifics in a variety of ways. One of the most widespread is chorusing, which consists in the repeated emission of the same calls by two or more individuals with frequent overlaps (as in chimpanzees *Pan troglodytes* [1] or colobus monkeys *Colobus guereza* [2], but also in insects and anurans [3]). Another is duetting, which generally involves more coordinated production of calls or songs between two mating individuals (especially in birds [4], but also in monogamous primates such as gibbons *Symphalangus syndactylus* [5]). Other vocal interactions found notably in non-human primates do not fall into these categories, such as what several authors call “conversation-like vocal exchanges” [6–9], sometimes also confusingly called antiphony [10] or antiphonal calling [11]. Indeed, these vocal exchanges typically concern only one specific call type of the species’ vocal repertoire, the so-called “contact call”. Contact calls do not occur at predictable times of the day or in the presence of a particular event but are emitted in several peaceful and affiliative contexts encountered throughout the day [7]. Furthermore, vocal exchanges of contact calls involve a diversity of recurrent partners belonging to the same social group and who can be of any age or sex (which differentiates them from choruses, which involve principally males, and from duets, being generally emitted by a mating pair). These partners are however not randomly selected: in several species, partners of contact call exchanges are also recurrent grooming partners suggesting an affiliative bonding functions (squirrel monkeys *Saimiri sciureus* [12], spider monkeys *Ateles geoffroyi* [13], lemurs *Lemur catta* [14], Japanese macaques *Macaca fuscata* [15], bonobos *Pan paniscus* [16]), in some other species elders individuals are preferred and receive more responses (Campbell’s monkeys *Cercopithecus campbelli* [17], common marmosets *Callithrix jacchus* [11]). These contact call exchanges are therefore often described as possible precursors to human conversations [7, 18], and the parallel is even more striking when it comes to their temporal structure qualified as “turn-taking” [6].

Turn-taking in human conversations consists in the smooth exchange of short utterances between several speakers that self-select themselves to intervene while avoiding overlaps and minimizing gaps between turns [19]. This overlap avoidance and gap minimization mechanism is a universal feature of human language despite some cultural variation in the response time due to languages differences [20], suggesting a biological basis. Turn-taking has thus been assessed in several non-human primates where the vast majority of individuals’ vocal responses has been found to occur within one to three seconds after a third party’s vocalization and to rarely overlap with it (less than one second for pygmy marmosets *Cebuella pygmaea* [8], squirrel monkeys *Saimiri sciureus* [21], Campbell’s monkeys *Cercopithecus campbelli* [17], Japanese macaques *Macaca fuscata* [22], up to three seconds for spider monkeys *Ateles geoffroyi* [13], red-capped mangabeys *Cercocebus torquatus* [23], western lowland gorillas *Gorilla gorilla gorilla* [24], and bonobos *Pan paniscus* [16]). Furthermore, this temporal structure can vary within a species. For example, in squirrel monkeys (*Saimiri sciureus*), response times are shorter between two individuals with high affinity compared to individuals with low affinity [12, 21]. In Japanese macaques (*macaca fuscata)*, response times can vary at two levels: within a group depending on the physical distance between vocal partners [25], and between different groups suggesting a cultural transmission [26].

Beyond a temporal pattern of vocal interaction, turn-taking could be a rule as in humans, implicitly known by the interactants and socially learned. Indeed, human turn-taking is learned early on during ontogeny by infants through the daily interactions they have with their parents [27–29]. This learning is fundamental because individuals who break this rule are perceived by their peers as being “less sociable” and having a “strong personality” [30, 31], which could impair their social relationships [32]. This may also be true for nonhuman primates. For example in marmosets (*Callithrix jacchus*), young individuals fail to use the appropriate type of vocalization for affiliative exchanges and interrupt others more than adults do [33]. This tendency decreases after a few months, with parent-guided social learning as they responded more to appropriately timed and chosen offspring vocalizations. A "violation-of-expectation" experimental paradigm can help demonstrate if turn-taking is a rule in nonhuman primates. The latter consists of observing the reactions of individuals to a stimulus assumed to follow the rule and comparing them to the reactions of the same individuals to a stimulus that break this hypothetical rule. A difference of reaction reveals that tested individuals have expectations on the stimuli, and so that what differ in these stimuli is a rule. In this line, two studies already showed that important features of turn-taking pattern act as a rule. Indeed, Lemasson et al [34] highlighted that adults Campbell’s monkeys (*Cercopithecus campbelli*) were more interested in exchanges with alternation (i.e. two interlocutors A and B call in turn to form ABA exchanges) than in exchanges with failed alternation (i.e. artificially rearranged BAA, consecutive calling from the same individuals being rare). Moreover, youngsters did not present such interest difference during the same experiment and were observed to interrupt more than adults during observations of spontaneous calling, suggesting that this rule need social learning to be mastered [34]. Using the same paradigm, Pougnault et al [35] highlighted that overlap avoidance matters in adults’ gorillas (*Gorilla gorilla gorilla*) as they paid less attention to the play-back of a vocal exchange with an overlap than to one that respect the species-specific response time. The intensity of the subject response also varied with subjects’ age, supporting again a possible role of learning.

These few studies, although interesting, remain rare and more comparative studies on a larger diversity of species is now needed. Red-capped mangabeys (*Cercocebus torquatus*) are interesting in this perspective because they have a well-known vocal repertoire and produce conversation-like contact call vocal exchanges [36]. A recent study indeed showed that red-capped mangabeys vocally respond within two-seconds to the vocalizations of a third party, with the most frequent response latency being 0.5 sec, and that social factors related to affinity and dominance of individuals shapes these latencies [23]. Here, we conducted an experimental study to assess whether turn-taking is a rule in mangabeys. We exposed captive adult males of different ages to vocal exchanges varying in temporal structure, constituted by calls from unknown individuals to neutralize the possibly confounding effect of the social bond between the subject and the stimulus individual [23]. We chose to broadcast the vocalizations of females, rather than males, because contact call rates (and thus exchange rates) are much more frequent in females than in males in that species [36]. The purpose of the experiment was to test the responses of individuals to regular vs irregular patterns. However, as this species forms multi-female multi-male groups with both sexes producing contact call exchanges [36], the temporal structure of these exchanges might be relevant as a conversational rule whatever the sex of the individuals involved. Three temporal structures were compared: a vocal exchange not violating any expectation with an usual response time (0.5 sec), a vocal exchange with turn-taking violation (call overlap) and a vocal exchange with an abnormally long delay (1.5 sec) to test for the first time the sensitivity to longer response times than usual. We expected subjects to be disinterested in unusual stimuli (here vocal exchanges with call overlap or abnormally long response time) as has been the case in other playback experiments [34, 35], and that this disinterest would be more pronounced among the more experienced individuals.

## Method

### Ethical note

This experimental but non-invasive study was conducted without modifying the living conditions of captive mangabeys housed at the Station Biologique de Paimpont (Brittany, France), where animal facilities and animal care procedures are regularly monitored by the responsible local authorities (Housing agreement for research D35211-18, delivered by the “Direction Départementale de la Cohésion Sociale et de la Protection des Populations” (DDCSPP)). This research protocol has been approved by the CREEA Ethic committee (“Comité Rennais d’Ethique en matière d’expérimentation animale”) under the reference APAFIS# 2021081711259974.

### Animals and housing conditions

Twelve captive adult male red-capped mangabeys (*Cercocebus torquatus*) were tested during this experiment. These individuals were all born in captivity and housed at the Station Biologique de Paimpont (Brittany, France). The animals were distributed in several enclosures during the experiments (**Table 1**): two individuals were alone (enclosures II, VI), one was living with two individuals from another species (*Cercopithecus campbelli*) (enclosure V), and the others were living with conspecifics in small groups (enclosures I, III, IV). Along the individuals’ life, group compositions changed several times. However, all individuals experienced living within social groups composed of both males and females for several years. The groups studied were not formed for this research program. They have lived in this primate facility since birth and will remain there until natural death (unless they are transferred from/to zoological parks as part of the EEP, a program to manage individuals of certain animal species present in European zoos for conservation purposes). This study is totally non-invasive, and the animals’ well-being is routinely monitored in accordance with the regulatory protocols for this type of animal facility. The enclosures for each group included an indoor part (from 8 to 27 m^2^) and an outdoor part (from 15 to 37m^2^), with a height ranging from 2.5 to 4m. Each indoor part was composed of three large adjacent cages. All subjects were in auditory and visual contact with one another outdoor as well as with females in nearby enclosures. Individuals were free to come and go in/out via tunnels, except during our experiment where the subject was isolated in the indoor part while all the other individuals were kept outside. All selected subjects were habituated to be temporarily isolated for cognitive experiments [37–39]. The indoor enclosure temperature was maintained at 22°C, and the floor was covered with straw and wood shavings, while the outdoor floor was made of cement or covered with bark. All spaces were enriched with wood and metal perches. The mangabeys were fed twice a day, with fresh fruits and vegetables in the morning and monkey chows in the afternoon. Water was available *ad libitum*.

**Table 1:** Characteristics of the adult males studied.

Enclosure	Subject	Age (years)
I.	Pirate	30
	George	16
	Carillon	15
	Elky	13
II.	Kamel	12
III.	Isba	18
	Roby	12
IV.	Tips	11
	Pouët	7
	Litchi	7
V.	Lupa	16
VI.	Coët	11

### Stimuli preparation

We created three different vocal exchange stimuli:

- 1^st^ condition “standard response time” (later named STANDARD): a likely vocal exchange between 2 individuals that respected the 500ms average inter-call duration of the species as showed in [23]
- 2^nd^ condition “long response time” (later named LONG): a rarer vocal exchange between 2 individuals that respected a long 1500ms inter-call duration
- 3^rd^ condition “call overlap” (later named OVERLAP): an unusual vocal exchange that violated the turn-taking rule with a 50% overlap between the 2 calls as in [35].

Each stimulus was composed of two contact calls (named “Ro” in that species [36]) from two different female callers. We did not use calls from mangabeys present in any of the enclosures to avoid other factors that could influence response time perception (i.e. dominance or affinity). We therefore selected calls from previous recordings of three individuals (A, B, C) no longer present on the site. We could thus build six different exchange dyads (AB, BA, BC, CB, AC, CA) and assigned them randomly to subjects. Each dyad was used to test two individuals. Calls were initially recorded with a Sony ECM-672 directional microphone connected to a Marantz PMD660 (44.1 kHz sampling rate—16 bit resolution). Only calls with good sound quality were selected (i.e. no echo, no background noise). A given call exemplar was never used twice for a given subject and when re-used for another subject, it was never placed in the same position (first or second) in the exchange. Vocal exchanges were created artificially stringing calls (with a silent gap between calls for STANDARD and LONG stimuli) using Audacity software version 2.4.2 [40]. For each call, linear fade-in and fade-out effects were applied (i.e. 100ms pre and 100ms post). Stimuli were homogenized in intensity so that they reach 60 db at one meter from the loudspeaker as in [35].

### Playback protocol

Each type of vocal exchanges was played back once to each mangabey subject using a Bluetooth loudspeaker JBL GO connected to a DELL Latitude 5400-NJVXR laptop. For each subject, we randomized the order of presentation of the three types of stimuli and we waited at least 5 days between two consecutive tests playback for a given individual to limit habituation effects. In a given half day, only one test was done per pair of adjacent enclosures. Prior to a playback, all non-tested individuals were moved outside by the caretaker who then hid the connected loudspeaker on the ground under the straw, next to a closed entrance trapdoor of the cage adjacent to the cage where the tested individual was. Prior to the test, the subject was distracted from the loudspeaker positioning process by the experimenter giving him cashew nuts, a highly appealing food. Then, the caretaker left and the subject stayed with the experimenter. The experimenter was sitting and gave irregularly cashew nuts to keep the tested mangabey in front of him with the loudspeaker in the subject’s back so that attentive responses to playbacks consisted in similar head orientation efforts for all subjects. When the subject was calm, not eating, with its face towards the experimenter and the surrounding environment was quiet, the playback was launched by the experimenter. During the whole playback session, the experimenter did not look at the mangabey so as not to influence its behavior. The experimenter looked at the screen of his camera JVC GZ-RX615BE to record the mangabey reaction for 60 seconds after the end of the second calls. At the end of these 60 seconds, the subject was rewarded independently of his reaction with cashew nuts. For each individual, we did a mock session with no sound diffusion (between the 1st and the 2nd playback, or between the 2nd and the 3rd playback) yet again to limit habituation.

### Data processing and analyses

Video recordings were analyzed using BORIS software [41]. We first extracted the 60 seconds to be analyzed (just after the second call of the stimulus) from each video so that, in a second step, the experimenter could code the samples blind to the condition being tested. Therefore, we analyzed N = 36 videos of 60 sec (12 males x 3 types of stimuli). We considered that a head orientation towards the loudspeaker was a behavior reflecting the individual’s interest for the playback as in [35, 42]. Given the initial position of the tested subjects, they had to turn their heads at least 90° to the side of the hidden loudspeaker so that it was in their visual field, and at a much greater angle if they turned in the opposite direction (also involving turning their bodies, or even moving). Coding via the BORIS software was conducted in slow motion, so that the observer could establish precisely when the loudspeaker was in the subject’s visual field, and thereby code the behavior. We measured two parameters of that behavior: the total duration (i.e. the time the individual observed the loudspeaker) and the occurrences (i.e. the number of times the individual observed the loudspeaker) as in [35]

Statistical analyses were conducted using R software version 4.3.2 [43]. We used Generalized Linear Mixed Models (GLMM) fit by a maximum likelihood to investigate link between mangabeys reactions to stimuli and age. We built two models, one with the total duration of head orientation toward the loudspeaker as dependent variable with truncated Poisson family distribution (because the total duration cannot exceed 60 sec), and the other model with the occurrences of head orientation toward the loudspeaker as dependent variable with Poisson family distribution. For both models, we took playback condition (three modalities: OVERLAP, STANDARD, LONG) and individual age (in year) as fixed factors in interaction, and the individual identity (N = 12) as well as playback exchange dyads (N = 6) as random factors. We ran our models using ‘glmmTMB’ R package version 1.1.8 [44] and we checked the model fits using the diagnostic plots generated by ‘DHARMa’ R package version 0.4.6 [45]. We analyzed these by conducting a type II ANOVA with a Wald chi-square test with ‘car’ R package version 3.1.2 [46], and did post-hoc tests with Tukey method for p-value adjustment using ‘lsmeans’ R package version 2.30.0 [47].

## Results

Subjects were interested in our stimuli but with important interindividual variations. During the 60 seconds following the end of the last call of each playback, they oriented their heads towards the loudspeaker 5.3 times on average (occurrences: mean = 5.3, median = 5, min = 1, max = 11), for a total duration of 15.3 sec on average (total duration: mean = 15.3 s, median = 13 s, min = 2 s, max = 42 s).

**Fig 1:**
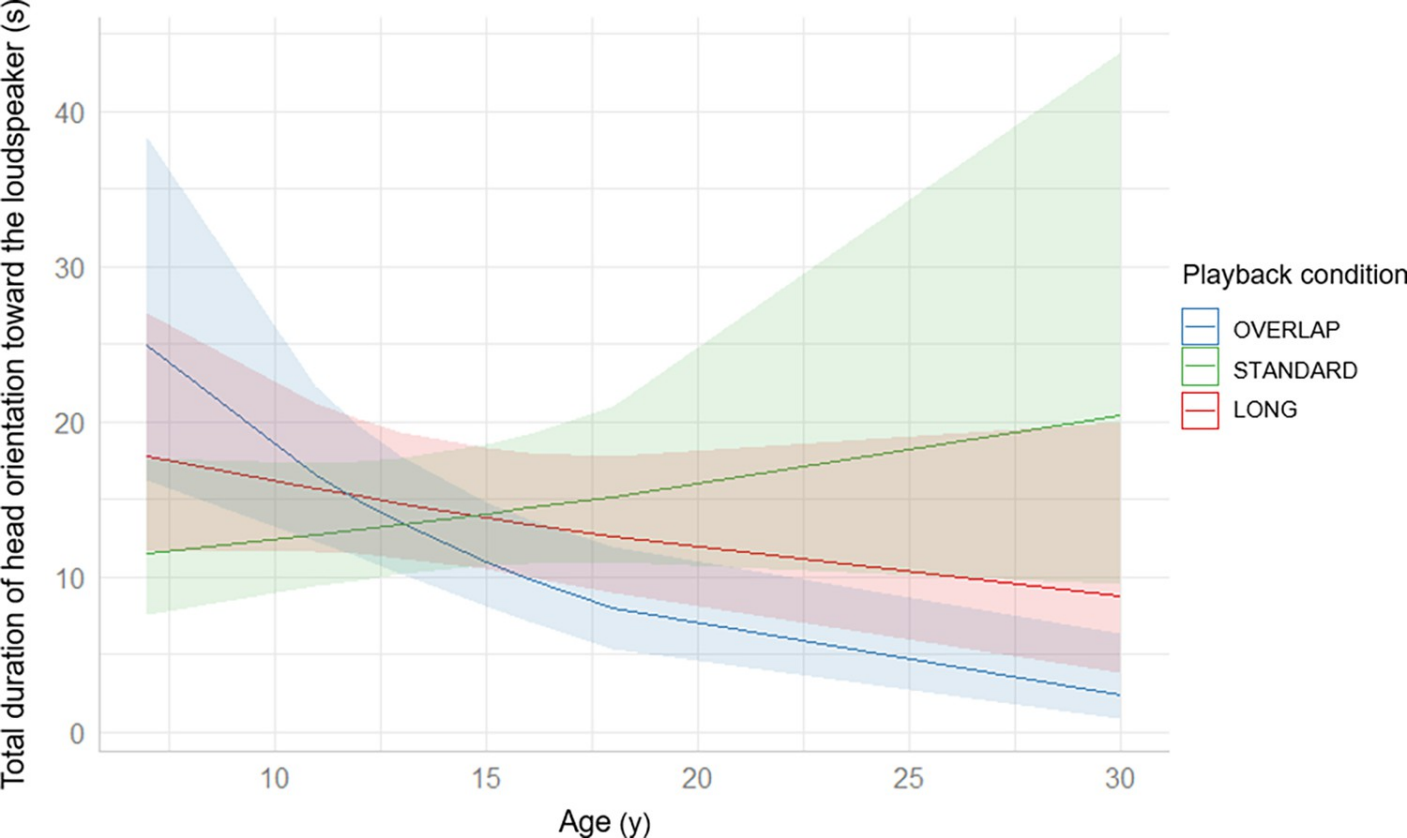
Marginal effects of the interaction between the total duration of head orientation towards the loudspeaker (s) and the age (y) of individuals. The lighter areas represent the confidence intervals for each curve. We plotted these marginal effects using “sjPlot” R package version 2.8.15 [48].

Analyses of both models (number of head orientations and duration) revealed that mangabeys reacted differently to playback conditions depending on their age. Concerning the total duration of head orientation toward the loudspeaker, the interaction between playback condition and age was indeed significant (GLMM: playback condition x age, Chi^2^ = 29.48, df = 2, p<0.001) whereas playback condition and age alone were not (GLMM: playback condition, Chi^2^ = 0.16, df = 2, p = 0.93; age, Chi^2^ = 0.41, df = 1, p = 0.52). Post-hoc Tukey tests highlighted that the three slopes (each slope representing the reaction of subjects to a playback condition according to their age, see **Fig 1**) significantly differed (LONG vs OVERLAP: estimate = 0.07, SE = 0.02, z-ratio = 2.98, p<0.01; LONG vs STANDARD: estimate = -0.06, SE = 0.02, z-ratio = -2.96, p<0.01; OVERLAP vs STANDARD: estimate = -0.13, SE = 0.02, z-ratio = -5.33, p<0.001). The slope corresponding to the OVERLAP condition is negative and significantly different from 0 (OVERLAP slope = -0.10, SE = 0.03, z-ratio = -3.62, p<0.001), which mean that the older an individual was, the less he oriented his head toward the loudspeaker (see **Fig 1**). The two other slopes did not significantly differ from 0 (LONG slope = -0.03, SE = 0.02, z-ratio = -1.26, p = 0.21; STANDARD slope = 0.02, SE = 0.02, z-ratio = 1.08, p = 0.28).

**Fig 2:**
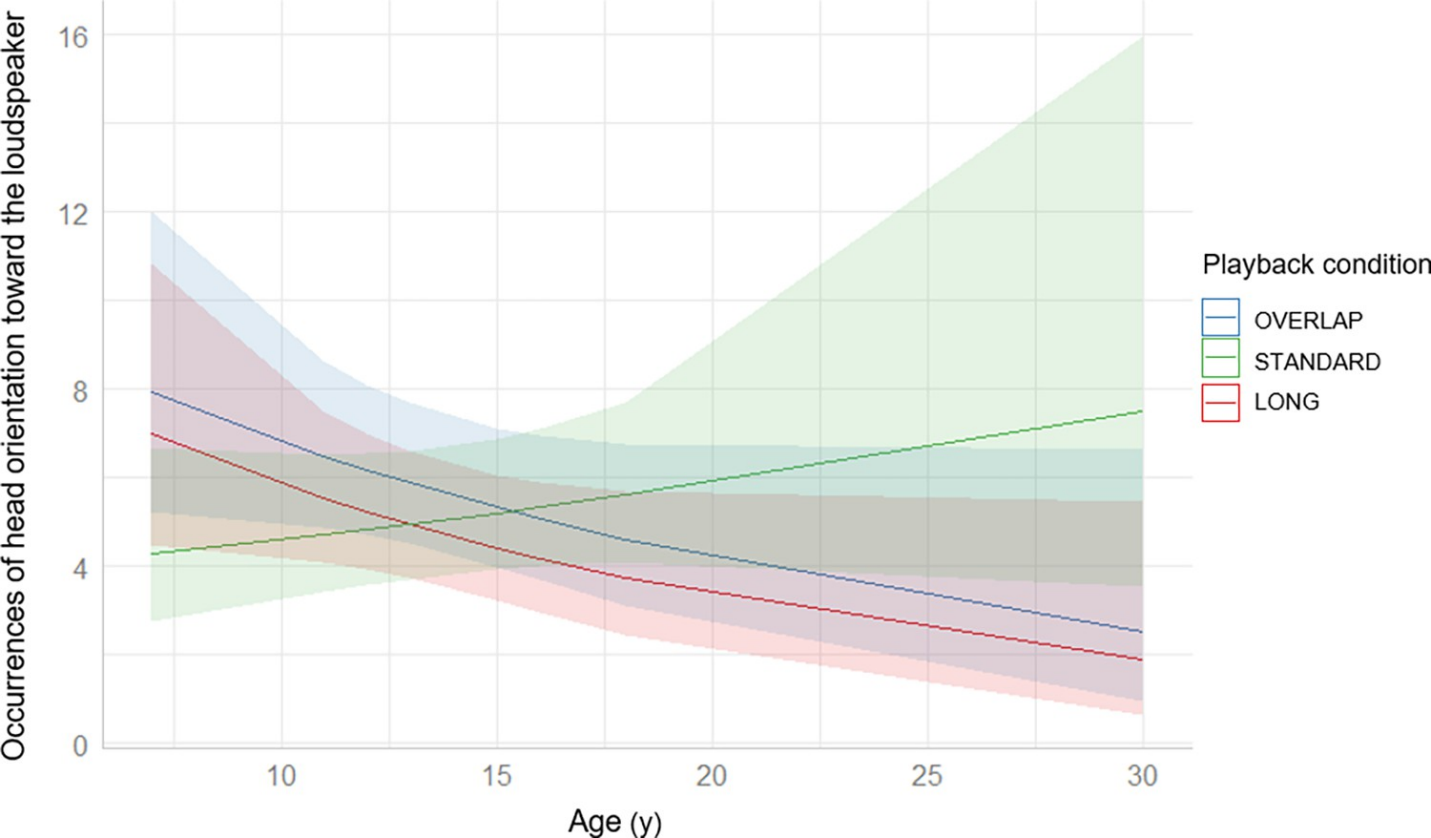
Marginal effects of the interaction between the occurrences of head orientation towards the loudspeaker (s) and the age (y) of individuals. The lighter areas represent the confidence intervals for each curve. We plotted these marginal effects using “sjPlot” R package version 2.8.15 [48].

Concerning the occurrences of head orientation toward the loudspeaker, the interaction between playback condition and age was here again significant (GLMM: playback condition x age, Chi^2^ = 7.80, df = 2, p = 0.02) and not the playback condition or the age when considered alone (GLMM: playback condition, Chi^2^ = 0.94, df = 2, p = 0.63; age, Chi^2^ = 0.93, df = 1, p = 0.33). Post-hoc Tukey showed that LONG and OVERLAP slopes are not significantly different (LONG vs OVERLAP: estimate = -0.01, SE = 0.04, z-ratio = -0.20, p = 0.98), but they both are or tend to be significantly different from STANDARD slope (LONG vs STANDARD: estimate = -0.08, SE = 0.03, z-ratio = -2.35, p = 0.05; OVERLAP vs STANDARD: estimate = -0.07, SE = 0.03, z-ratio = -2.29, p = 0.06). LONG and OVERLAP slopes also tend to be significantly different from 0 (LONG slope = -0.06, SE = 0.03, z-ratio = -1.89, p = 0.06; OVERLAP slope = -0.05, SE = 0.03, z-ratio = -1.80, p = 0.07), while STANDARD slope is not (STANDARD slope = 0.02, SE = 0.02, z-ratio = 1.05, p = 0.3). Since the formers are negative, this means that the older an individual, the fewer occurrences of head orientation towards the loudspeaker in the LONG and OVERLAP conditions (see **Fig 2**).

## Discussion

As we predicted, subjects’ interest toward the stimuli varied according to the temporal pattern of vocal exchanges played back as a function of age. We used two measures to assess this interest: the total duration and the occurrences of head orientation toward the loudspeaker. Our analyses showed that the older (and thus the more socially experienced) an individual was, the less the interested showed in a vocal exchange with an unusual overlap or an abnormally long response time.

These findings are in line with those of other studies suggesting the existence of conversation rules in several non-human primate species, which can be characterized as follows: caller alternation [34], overlap avoidance [35], acoustic matching [42], calling order [49]. Here, we showed for the first time in nonhuman primates the importance of another conversational rule: response time minimization. Until now, this had only been described in observational studies in non-human and human primates [20]. Minimizing response times is far from trivial for humans since it imposes a significant constraint on language production. Levinson & Torreira [50] has indeed pointed out that the average response time present in human conversations is much shorter than the time needed to plan the production of a word, forcing interlocutors to start planning during the previous interlocutor’s turn. This is what human children struggle for, as they take more time to respond linguistically than adults [28]. The challenge of integrating language into turn-taking communication raises questions about the evolutionary pressures that have led to this interactional niche underlying language. In this respect, demonstrating that adult non-human primates might be less interested in vocal exchanges with longer response times is a first step to understand such evolutionary pressures. Motivational factors can be put forward, based on the evidence that vocal exchanges facilitate the creation of affiliative social bonds: individuals would therefore be motivated to respond immediately to create a bond with a given caller (before another individual does so), and a rapidly occurring response could therefore be indicative of this motivation to bond [23].

Obviously, our results indicate that age was a modulating factor in our subjects’ interest about more or less well-structured vocal exchanges. In our models, the playback condition was indeed not significant by itself, but only in interaction with age. Here, older males were more disinterested in the incongruent situations than younger males. The fact that an older age, and thus social experience, conditions this disinterest is consistent with the idea of an acquisition by social learning of the rule. Several conversational rules were already suggested to be socially learned, such as the “acoustic matching” rule (i.e. the responder adjusts the acoustic structure of his call to copy the initiator of the exchange) in spider monkeys and Japanese macaques [13, 42], and the “turn-taking” rule in Campbell’s monkeys and gorillas [34, 35]. These evidence are far from trivial due to the historical debate on vocal social learning in nonhuman primates [51], but also because social learning is an inherent feature of what we call a “rule”. Moreover, our results showed differences between young adults and older ones (as in gorillas [35]), suggesting that social experience continues to play a role beyond youth throughout an individual’s life.

Thus, this study showed that red-capped mangabeys had expectations about the temporal pattern of vocal exchanges heard based on their social experience, suggesting that turn-taking is a rule for this species.
